# Protocells: At the Interface of Life and Non-Life

**DOI:** 10.3390/life5010447

**Published:** 2015-02-09

**Authors:** Wentao Ma, Yu Feng

**Affiliations:** College of Life Sciences, Wuhan University, Wuhan 430072, China; E-Mail: fengyu@whu.edu.cn

**Keywords:** the essence of life, RNA-based protocells, the RNA world, artificial cells, synthetic biology

## Abstract

The cellular form, manifesting as a membrane-bounded system (comprising various functional molecules), is essential to life. The ultimate reason for this is that, typically, one functional molecule can only adopt one “correct” structure to perform one special function (e.g., an enzyme), and thus molecular cooperation is inevitable. While this is particularly true for advanced life with complex functions, it should have already been true for life at its outset with only limited functions, which entailed some sort of primitive cellular form—“protocells”. At the very beginning, the protocells may have even been unable to intervene in the growth of their own membrane, which can be called “pseudo-protocells”. Then, the ability to synthesize membrane components (amphiphiles) may have emerged under selective pressure, leading to “true-protocells”. The emergence of a “chromosome” (with genes linked together)—thus avoiding “gene-loss” during the protocell division, was another key event in the evolution of protocells. Such “unitary-protocells”, containing a central genetic molecule, may have appeared as a milestone—in principle, since then life could evolve endlessly, “gaining” more and more functions by introducing new genes. To synthesize in laboratory these different types of protocells, which stand at the interface between life and non-life, would greatly enhance our understanding on the essence of life.

## 1. Why Is Life Based on the Cellular Form?

Cells are fundamental to life nowadays. All living things are made up of cells, except for viruses, which, though capable of existing “physically” independent of cells, are only “biologically” active within host cells: but why? A general answer is that living activities are based on the cooperation of molecules, and lipid vesicles, which are bounded by amphiphilic membranes in water, provide the most effective form in nature for such cooperation. However, to provide a more exact answer we need a more detailed analysis. Does life really have to be implemented by the cooperation of molecules? Moreover, if yes, how about other forms for such molecular cooperation? In the following, we will see that such an analysis would lead us to inquire into the very early stages of life.

Though there is no consensus on a definition for life, there is no doubt that life is something capable of Darwinian evolution [1,2,3]. Therefore, there is also no doubt that life should comprise two key features: the genetic and the functional, which make natural selection, and thus Darwinian evolution, possible. In the modern life forms, the former feature is implemented mainly by DNA and the latter mainly by proteins. Then the cooperation of these two types of molecule seems inevitable. However, while it is well known that RNA can act as genetic material in some viruses, it became clear several decades ago that RNA can also implement the functional feature [4,5]. The notion that RNA can implement both the two key features bred the now famous RNA world hypothesis [6,7]. The hypothesis asserts that life might have ever been based solely on RNA in its early history, perhaps at the very beginning.

For the topic about the arising of life from non-life, the RNA world hypothesis helps to sidestep the “which came first, DNA or protein?” dilemma in the context that both the genetic feature and the functional feature are indispensable for life. Regarding the topic here, it means that the cooperation of “two types of molecule” could be “avoided” in life. Moreover, it has even been postulated that the RNA world might have begun with some kind of “RNA replicase” (ribozyme), which can exhibit both those two key features by catalyzing its own replication [8,9,10]. This implies that the cooperation of “two kinds of molecule” can also be avoided, provided that the RNA replicase and its complementary sequence are deemed as the same kind. However, even in this simplest scenario, the cooperation between two “individual” molecules cannot be avoided: actually, in such replication, one molecule (the RNA replicase) would act as a ribozyme, and the other (the RNA replicase or its complementary chain) as a template. To examine the question thoroughly, we can imagine an extreme case: an “intramolecular” RNA replicase [11], during whose replication one of the two domains of this RNA molecule may fold back to catalyze the copying of the other domain, and then *vice versa*. However, even if such an extreme scenario involving intramolecular action was possible in the very beginning of life, it was not possible for the life later—perhaps already impossible only a short time later when a second function appeared. Can one imagine a large RNA molecule with quite a few domains to implement different functions respectively? In other words, even if the two key features, genetic and functional, could be implemented on a single molecule at the very beginning (while there was only one function—the catalysis of template-directed synthesis of RNA), when life evolved forwards and different functional molecules (e.g., various ribozymes in the RNA world) emerged, the cooperation between molecules would have become inevitable. 

The key point is: a functional molecule (either a protein or an RNA) typically has only one function (two occasionally and at most several in some rare cases). Therefore, given that more and more functions would emerge through evolution, more and more functional molecules would become involved in the living activities of organisms. Indeed, molecular cooperation would not only be indispensable but also become more and more complicated.

Then, obviously, there is a problem of spatial limitation—in which way could these molecules be kept sufficiently close to each other to ensure such cooperation? Several ways have been proposed for such spatial limitation at an early stage. One way is by the adsorption of mineral surface. For example, the RNA world, with its cooperative molecules, is speculated to have begun on the surface of minerals such as montmorillonite and hydroxylapatite [12,13,14,15,16]. A second way involves the freezing phase of ice-water, which has also been suggested to be a possible circumstance for the early RNA world [17,18,19,20,21,22,23]. Freezing provides compartmentalization for the RNA molecules (in water solution) trapped in the ice crystal, and thus prevents their dispersal [17,18,23]. A similar idea involves the porous structure of rocks in alkaline thermal vents on the sea floor, which have been suggested as the hatchery of life [24,25,26]. In this scenario, RNA (and later RNA/protein) molecules collaborate in pores bounded by rocky walls, dubbed “inorganic cells”. Regarding the way of mineral surface, an apparent problem is that with the emergence of more functional molecules, this manner of spatial limitation would become insufficient. For the latter two ways, the “fixed room” strategy, either with icy or rocky walls, would greatly restrict the migration/distribution of life during evolution. So then, the genuine cellular form, with genetic and functional molecules bounded by lipid membrane composed of amphiphilic molecules, making independent free-living entities of molecular assembly, seems to be the most natural choice for life in its development. Notably, the formation of the lipid vesicles may have been a natural process in prebiotic environments [27,28,29,30], and genetic polymers could have been brought naturally into the interior of the vesicles or have been engendered naturally inside the vesicles [29,30,31,32,33]. Therefore, the so-called genuine cellular form may have emerged rather early.

## 2. Protocells: The Primitive Cellular Form

Modern cells are very complicated. The simplest free-living organism/cell in nature known to date is *Mycoplasma genitalium*, which has approximately 470 (protein-coding) genes [34]. Starting with this tiny cell and some other small organisms whose genomes have been sequenced, researchers have tried to identify the “minimal cell” capable of self-sustaining by approaches like searching the common genes among different genomes and analyzing viable gene knockouts. Their results indicate a minimum scale of approximately 200~250 genes [35,36,37]. If the lifestyle is not a constraint, the simplest cell in nature known to date is a bacterial endosymbiont found in insects, *Tremblaya princeps* PCVAL which has still 116 genes [38] (Note: if the genome size is adopted as the criterion, the record falls on *Nasuia deltocephalinicola*, which itself has 137 genes [39]). As mentioned above, the cellular form is the natural choice for the development of life and likely to have emerged very early—to ensure molecular cooperation. Obviously, at the very beginning, the cellular form could not have been so complicated; otherwise, it would be difficult to interpret how the cellular form could have first emerged. Therefore, there must have been some much simpler cellular form(s), which have been referred to as “primitive cells” or “protocells” [29,30,40,41].

As it was stated, though there are as yet a number of obstacles for the RNA world hypothesis, it remains the best, central idea in our efforts to interpret the origin of life [42]. According to the RNA world hypothesis, the functions responsible for translation, as well as those associated with DNA, might not have existed at the early stage of life. Thus, the gene number could be reduced significantly. In fact, in the exploration of simple cellular forms, the Minimal Cell project represented the top-down approach [43], and on the other end there was another project called Synthesizing Life, representing the bottom-up approach [44]. The Synthesizing Life project is just based on the concept of the “RNA-based protocell”. For example, it is thought that the simplest cell could just be a lipid vesicle containing a sort of RNA replicase [44]. The membrane, composed of amphiphilic molecules, provides a boundary to ensure the cooperation between the RNA replicase molecules—as mentioned above already, in the case of such cooperation, one acting as a ribozyme and the other as a template. This line of investigation has brought us quite a few steps forward, especially in respect to RNA synthesis in lipid vesicles [29,45], but hurdles remain concerning RNA-catalyzed RNA replication—our long-standing attempt to construct a real RNA replicase, which can catalyze its own replication, has not succeeded to date [46,47,48,49,50].

Whether or not the lipid vesicle containing the RNA replicase can represent the simplest cellular form, the notion of the RNA-based protocell serves well as a “working concept” for us to examine some fundamental issues concerning the primitive cellular form. As we will see in the analysis, we can define different stages for the protocells. Note that here our main aim is to deduce how the primitive cellular form, *i.e.*, protocells, may have evolved. We rely heavily on the RNA world hypothesis because to date it is the only idea put forth that does not evade the question of how Darwinian evolution could have got start, which is associated with the topic here—the interface of life and non-life. If the following discussion, which adopts the notion of the RNA-based protocell, appears to be delineating a detailed scenario, one should keep in mind that it just represents one speculation regarding the early stage in the origin of life. For example, if there was some “pre-RNA world(s)” that used RNA-like molecules as genetic and functional material [7,10], the protocells at the very beginning should have been based on the RNA-like molecules instead. As another example, if those “inorganic cells” could have evolved to a rather complex level before the emergence of lipid membranes, as suggested in the “alkaline thermal vents” hypothesis [25,26], the scenario would have been completely different.

## 3. Pseudo-Protocells and True-Protocells: About the Membrane Synthesis

First, let us look into the “reproduction” of the protocells. Reproduction is a prerequisite for Darwinian evolution. If a single molecule is deemed as a “living unit” (e.g., an RNA replicase as a candidate), its replication is just such “reproduction”. However, the reproduction of a molecular assembly like that in a cellular form is no doubt more complex. It includes the growth of the “molecular assembly”, *i.e.*, the duplication of the molecules therein, and then the fission. For modern cells, both growth and fission are well controlled, involving many genes/functions. However, for those simplest protocells, which comprise few genes/functions, how could these be carried out? Primordially, the division of a protocell may have occurred spontaneously, *i.e.*, by physical mechanisms, when it grows to an unstable level [44,51]. The main problem is associated with growth. Cellular growth requires a coupled growth of the core (for an RNA-based protocell, the RNA molecules enclosed within) and membrane (composed of amphiphilic molecules). At such a primitive stage, how could this coupled growth be achieved? This is an essential issue for these simple protocells, arising from the most fundamental level in concept. Indeed, physical mechanisms are, again, likely to have provided the solution.

In a distinctive study, it was observed that fatty acid vesicles encompassing RNA molecules were more swollen (under the osmotic pressure) and would grow to a larger size by sacrificing empty vesicles, which were more relaxed [52] (note: while the modern cell membrane is mainly composed of phospholipids, the protocell membrane is likely to have been composed of a simpler type of amphiphilic molecule, fatty acids, especially at the very beginning [30,41]). The underlying mechanism should be that the amphiphiles in the membrane of a more swollen vesicle are less “willing” to leave the membrane, and thus the RNA-containing vesicles would win the competition in the routine exchange of amphiphiles between the vesicles (via the circumambient solution) [52]. That is to say, the increase of RNA molecules within a protocell might have brought about the growth of the membrane this way, and finally the reproduction via division. Indeed, for the simplest protocells, e.g., the protocells containing only an RNA replicase, this was a handy (if not the only) mechanism they could exploit. Because such protocells lacked a function that dealt with their own membrane synthesis, we call them “pseudo-protocells” [53].

**Figure 1:**
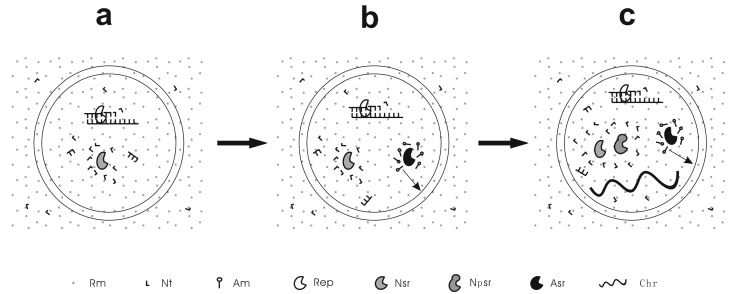
A scheme to exemplify the different stages of the RNA-based protocells. (**a**) Pseudo-protocell, the protocell lacking the capability to synthesize its own membrane; (**b**) True-protocell, the protocell processing a membrane synthesis function; (**c**) Unitary-protocell, the protocell holding a primordial RNA chromosome as the central genetic molecule and thus acting integrally as a unit for natural selection. Rm = raw material; Nt = nucleotide; Am = amphiphile; Rep = RNA replicase; Nsr = nucleotide synthetase ribozyme; Npsr = nucleotide precursor synthetase ribozyme; Asr = amphiphile synthetase ribozyme; Chr = chromosome. The arrow means that the amphiphiles may join the membrane of the protocell. Note: Nsr and Npsr represent different ribozymes that participate in different nucleotide synthesis steps.

The pseudo-protocells might have developed to a more complex level than containing only an RNA replicase, e.g., incorporating a ribozyme catalyzing the synthesis of nucleotides (the nucleotide synthetase ribozyme) [53] (Figure 1A). However, the emergence of a membrane synthesis function should already have been “in urgent need” by then because if only abiotic pathways were relied on, the shortage of membrane components would seriously limit the proliferation and spread of the protocells. Could natural selection have worked to bring about such a function? Indeed, a relevant mechanism has been suggested [54]. An amphiphile synthetase ribozyme could favor membrane growth (note that the membrane would grow by naturally incorporating new amphiphilic components), and thus bring about an increase in cellular space under the membrane tension caused by the osmotic pressure mentioned above [52]. Raw materials, e.g., nucleotide precursors, in the protocell would then be diluted, leading to a further influx of the raw materials (owing to the concentration equilibrium flanking the membrane) and thus favoring the synthesis of RNA inside the protocell. Instead of being triggered by core-growth as in the pseudo-protocells, in this type of core-membrane coupling, the membrane growth brings about core growth. That is, some sort of amphiphile synthetase ribozyme could have arisen under Darwinian selection—the protocells possessing the ribozyme were superior to those without it on account of the availability of those raw materials. This hypothesis is supported by subsequent computer simulations—an amphiphile synthetase ribozyme, along with the protocells containing this ribozyme, could proliferate and spread in the model system [53,54]. In contrast to “pseudo-protocells”, we call these protocells, already possessing a membrane synthesis function, “true-protocells” (Figure 1B) [53].

## 4. Unitary-Protocells: Linked Genes and the Starting Point of Real Life

A true-protocell may have contained a few different functional RNAs, e.g., an RNA replicase, a ribozyme catalyzing the synthesis of nucleotides, a ribozyme catalyzing the synthesis of membrane components, *etc.* However, this so-called “bag of genes” faced a problem [55] when the protocell had grown to a sufficient size and divided spontaneously (as mentioned above), the RNA molecules therein would have been distributed randomly to the daughter protocells. So then, there would have been a risk of “gene loss”—a daughter protocell may have not inherited even a single copy of certain functional RNA. When more functional RNAs emerged during evolution, the problem would become more serious—it is likely that an offspring protocell would lack some genes, these or those, more or less. A direct solution to this problem is the emergence of a “chromosome” [55,56]. When the genes were linked together as a “chromosome”, being distributed together, the risk of gene loss would be released. Additionally, the emergence of the chromosome might, potentially, provide a solution to the dilemma of different structural preferences for the genetic and functional polymers by labor division: the RNA chromosome would shoulder the genetic task specially (thus, could be looser in structure to be a better template in replication) whereas the ribozymes work specially as functional molecules (thus, could bear stronger structures to be more efficient) [57].

However, there would have been quite a few difficulties for the emergence of the primordial chromosome, e.g., the RNA chromosome would have been much longer than single RNA genes and hence run a greater risk of degradation (chain breaking) before completing replication; the copying of the chromosome may have started at sites in the middle of the chain, resulting in a partial replication; without a complex transcription mechanism (e.g., with promoters and ending signals), the synthesis of distinct gene products (*i.e.*, the ribozymes) from the chromosome would have become problematic. In a recent paper, inspired by features of viroids [58,59,60], we postulated that circularity plus self-cleavage may have been adopted as a strategy by the primordial RNA chromosome to overcome these difficulties, and this idea was supported by the subsequent analysis of relevant evolutionary dynamics by computer simulation [57]. The strategy is very simple and likely to have been used in the RNA world, as an early stage in evolution.

Parallel to the terms “pseudo-protocells” and “true-protocells”, we would like to call the protocells containing a primordial chromosome “unitary-protocells” (Figure 1C). By “unitary” we imply that such a protocell, holding a central genetic molecule, acted integrally as a unit for natural selection. Indeed, if we refer to a unit for natural selection as a “Darwinian entity” [61], it was only after the emergence of the unitary-protocells that the genuine Darwinian entities at cellular level arose. In fact, we could use more straightforward remarks to emphasize the great significance of this type of protocell: given that Darwinian entities in the form of individual molecules (e.g., some kind of RNA replicase ribozyme), if existing at all, were “futureless” in the subsequent evolution (toward complexity), with regard to acting integrally up against natural selection, the “unitary-protocells” represented the ultimate choice for life’s realization in the physico-chemical world. Let us explain this assertion in more detail.

On the genetic feature aspect, which is realized via the template-directed replication by the “base-pairing mechanism”, there is no serious problem to accommodate more functions, because different “genes” may be replicated sequentially in the same manner in a single larger molecule. However, on the functional feature aspect, which is realized by the folding of the functional polymer into a special structure, things are completely different. As mentioned above, a functional molecule typically carries out only one function. We could not imagine a large functional molecule (either RNA or protein) with a number of domains to implement a number of different functions respectively. Moreover, we could not expect one functional polymer, either RNA or protein, to be capable of carrying out many different tasks in different circumstances respectively. These are based on the constraints imposed by the physico-chemical rules governing the folding of the macromolecules in water. It is unlikely that the different domains in that large functional molecule could fold into their “correct” structures without interfering with each other, and a functional molecule with a definite sequence could not have different “correct” structures (*i.e.*, the so-called rule of “the primary structure determining the tertiary structure”). In a word, typically one sequence has one function because typically one sequence has one structure and typically one structure has one function. Indeed, molecular cooperation was inevitable only if more functions “ought to” appear in evolution. Fortunately, also in this physico-chemical world, amphiphilic molecules can assemble to form vesicles in water, providing a simple and effective way to ensure such molecular cooperation.

Therefore, the unitary-protocell, gathering all genes into one central genetic molecule but dispersing corresponding functions into different functional molecules, and bounded by the lipid membrane, should have represented the most natural, the most parsimonious and the ultimate form for life’s realization in the physico-chemical world. In a more vivid description: when more functions evolve, though a single molecule can still carry the genetic feature by itself, it has to “express” its function feature into different functional molecules and consequently, has to seek an effective way to hold the functional molecules close to itself. Indeed, this organizational form has lasted to the emergence of modern cells—a prokaryotic cell has only one chromosome, which codes for quite a lot of functional molecules. The emergence of multiple chromosomes in eukaryotic cells was brought about by the necessity to accelerate whole genome replication, when genetic information became vast in quantity—*i.e.*, replication can be carried out simultaneously on different chromosomes. This would have been possible only when the cellular form became sufficiently sophisticated and the cell division could be well controlled by the cell itself—the problem of “gene loss” no longer exists: the chromosomes are precisely distributed into two daughter cells as two identical groups. 

If life should be something capable of evolving endlessly [62], the unitary-protocells may have represented the starting point of life, because in principle, since then more and more functions could appear simply through the introduction of additional genes into the central genetic molecule. In fact, with regard to the organizational form of Darwinian entities, there is no essential distinction between such unitary-protocells and modern life. This is a milestone. For instance, according to the scenario of the RNA world, DNA and proteins would emerge later, replacing RNA as (major) genetic material and (major) functional material respectively, then—in some sense, even these “revolutionary” events could not match the significance of the emergence of the so-called “unitary-protocells”.

## 5. Understanding Life by Synthesizing Protocells

Modern biology has achieved marvelous progresses since the rise of molecular biology, which was marked by the discovery of the double helix structure of DNA in the middle of the last century [63]. Today such a success has been further pushed forward by the growth of genomics and the appearance of systems biology. Indeed, we now know a considerable amount about the “secret” of life [64]. However, somewhat ironically, we are still quite uncertain about the “essence” of life. Although the question of “what is life?” has been discussed for a long time and in many occasions (e.g., it is the title of that famous pamphlet of Schrodinger [65], which was published even earlier than the discovery of the DNA double helix), we are still unable to formulate a formal definition that does not incite debate [3,66]. Now, finding a satisfactory solution to this problem represents one of the greatest challenges to us all: for the so-called “life-science” discipline and even for the natural sciences in general.

A logical way to deal with this challenge is to investigate the interface between life and non-life, which by definition, is relevant to the field of the origin of life. According to our analysis here, at the outset of life, spatial limitation to ensure molecular cooperation was already quite significant when there were only limited functions, and protocells likely emerged rather early. In other words, it was just the protocells that stood at this interface. However, because the origin of life, as a historical process that occurred in the remote past, bears predestined uncertainties, we should pay more attention to the scientific aspect, which pursues repeatable facts and relevant rules [61]. So a good strategy is to “synthesize” these protocells in laboratory. If we could really construct in laboratory such protocells (more concretely, the RNA-based protocells [44,67]), undoubtedly, we would make great progress in understanding the essence of life [68,69,70,71,72]. In light of our conceptual analysis, we can go ahead with our attempts to synthesize pseudo-protocells, true-protocells, and finally, the unitary-protocells. Particularly, if we can ultimately synthesize the unitary-protocells, that is, the protocells containing a central genetic molecule (*i.e.*, a primordial chromosome with linked genes), we may feel that we obtain a “snapshot” concerning the starting point of real life.
